# Passing Events and Topics

**Published:** 1920-11-06

**Authors:** 


### Passing Events and Topics.

### New Secretary at Paddington Green.

The new Secretary of Paddington Green Hospital is Mr. J. A. Hamblin, who was recently Secretary of the inoculation department at St. Mary's Hospital, Paddington.

### British Hospital for Incurables.

The Marchioness of Carisbrooke on October 27 visited the British Home and Hospital for Incurables at Streatham, and opened a sale of the patients' work, the results of which will be devoted exclusively for their benefit. She was presented with a bouquet by the oldest inmate.

### Great Eye Hospital in Difficulties.

Everybody knows "Moorfields," as the Royal London Ophthalmic Hospital is still called, although it has been in the City Road for many years. It is the oldest hospital of its kind; and the patients are very numerous. The hospital needs £100,000, and if the economic significance of the preservation of eyesight can be brought home to the public all that is required should cheerfully be given.

Princess Louise, Duchess of Argyll, has been pleased to become President of the Ladies' Association of the Great Northern Central Hospital, which was the first London hospital to form such a body. The Association, which celebrated its Jubilee last year, has been a pioneer in many ways in the advancement and efficiency of the institution. Next May the Association will hold a bazaar in the West End in aid of the Nurses' Home Fund.

